# Longitudinal body mass index trajectories at preschool age: children with rapid growth have differential composition of the gut microbiota in the first year of life

**DOI:** 10.1038/s41366-022-01117-z

**Published:** 2022-04-15

**Authors:** Myrtha E. Reyna, Charisse Petersen, Darlene L. Y. Dai, Ruixue Dai, Allan B. Becker, Meghan B. Azad, Kozeta Miliku, Diana L. Lefebvre, Theo J. Moraes, Piushkumar J. Mandhane, Rozlyn C. T. Boutin, B. Brett Finlay, Elinor Simons, Anita L. Kozyrskyj, Wendy Lou, Stuart E. Turvey, Padmaja Subbarao

**Affiliations:** 1 Translational Medicine Program, Department of Pediatrics, The Hospital for Sick Children, Toronto, ON Canada; 2 Department of Pediatrics, BC Children’s Hospital, University of British Columbia, Vancouver, BC Canada; 3 Section of Allergy and Immunology, Department of Pediatrics and Child Health, University of Manitoba, Winnipeg, MB Canada; 4 Developmental Origins of Chronic Diseases in Children Network (DEVOTION), Children’s Hospital, Winnipeg, MB Canada; 5 Department of Food and Human Nutritional Sciences, University of Manitoba, Winnipeg, MB Canada; 6 Department of Medicine, McMaster University, Hamilton, ON Canada; 7 Department of Pediatrics, University of Alberta, Edmonton, AB Canada; 8 Department of Microbiology and Immunology, University of British Columbia, Vancouver, BC Canada; 9 Michael Smith Laboratories, UBC, Vancouver, BC Canada; 10 Department of Biochemistry and Molecular Biology, University of British Columbia, Vancouver, BC Canada; 11 Dalla Lana School of Public Health, University of Toronto, Toronto, ON Canada

**Keywords:** Translational research, Paediatrics

## Abstract

**Background/Objective:**

The steep rise in childhood obesity has emerged as a worldwide public health problem. The first 4 years of life are a critical window where long-term developmental patterns of body mass index (BMI) are established and a critical period for microbiota maturation. Understanding how the early-life microbiota relate to preschool growth may be useful for identifying preventive interventions for childhood obesity. We aim to investigate whether longitudinal shifts within the bacterial community between 3 months and 1 year of life are associated with preschool BMI *z*-score trajectories.

**Methods:**

BMI trajectories from birth to 5 years of age were identified using group-based trajectory modeling in 3059 children. Their association with familial and environmental factors were analyzed. Infant gut microbiota at 3 months and 1 year was defined by 16S RNA sequencing and changes in diversity and composition within each BMIz trajectory were analyzed.

**Results:**

Four BMIz trajectories were identified: low stable, normative, high stable, and rapid growth. Infants in the rapid growth trajectory were less likely to have been breastfed, and gained less microbiota diversity in the first year of life. Relative abundance of *Akkermansia* increased with age in children with stable growth, but decreased in those with rapid growth, abundance of *Ruminococcus* and *Clostridium* at 1 year were elevated in children with rapid growth. Children who were breastfed at 6 months had increased levels of *Sutterella*, and decreased levels of *Ruminococcus* and *Clostridium*.

**Conclusion:**

This study provides new insights into the relationship between the gut microbiota in infancy and patterns of growth in a cohort of preschool Canadian children. We highlight that rapid growth since birth is associated with bacteria shown in animal models to have a causative role in weight gain. Our findings support a novel avenue of research targeted on tangible interventions to reduce childhood obesity.

## Introduction

The steep rise in rates of obesity in the last decades has emerged as a worldwide public health problem from which children are not exempt [1]. Obesity as early as 6 months of age has been reported to track into school age [2], and has been linked to increased risk of many adverse health conditions in adolescence and adulthood including cardiometabolic, pulmonary, and psychological disorders [3–6].

The collective consortia of resident bacteria and their functional genetic capacity making up the microbiota and microbiome, respectively, is a key regulator of host metabolism and obesity. With an estimated 10^12^ bacteria residing along our digestive tract, the microbiota both directly shapes nutrient availability within the gut as well as regulates host metabolic pathways responsible for transporting and storing those nutrients [7–10]. A number of studies have investigated the role of the microbiota in obesity in adult humans and animal models, even going so far as to develop weight loss therapies using microbial candidates such as *Akkermansia muciniphila* [11–14]. However, studies focusing on children are limited to cross-sectional indicators of obesity, and only a few analyzed the relationship between the gut microbiota and longitudinal changes in growth [15].

Longitudinal studies of childhood growth are essential due to the individual variations in patterns of weight over time, and have been shown to have better predictive value over cross-sectional measures for future body composition and obesity [16]. Furthermore, obesity in the first years of life has been associated to genetic and early-life environmental factors such as pre-pregnancy maternal body mass index, gestational weight gain, feeding practices (i.e., breastfeeding and formula use), and socioeconomic adversity [17, 18]. Early life also represents a critical window for microbiota maturation, when exogenous factors such as mode of delivery, diet, antibiotics exposure, and built environment heavily influence bacterial membership and composition within the gut [19]. This results in a dramatic expansion of diversity as well as fluctuations in bacterial colonization that stabilizes after 1–3 years of age [19, 20]. The early-life microbiota has been associated with a number of disorders that appear later in childhood, however only a few studies have focused on longitudinal changes within the infant bacterial community. Since transitions within the infant diet also occur during this time (e.g., solid food introduction and reduction of breastmilk/formula), understanding how shifts within the early-life microbiota relate to rapid growth or obesity may be particularly useful for identifying potential preventive interventions.

This study will investigate whether BMIz trajectories of growth up to age 5 years are associated with longitudinal shifts within the bacterial community between 3 months and 1 year of life. We used a data-driven approach to develop longitudinal preschool BMIz trajectories in children enrolled in the CHILD Cohort Study. In a subsample of the cohort, we quantified changes in the infant gut microbiota diversity and composition and determined whether these changes were associated with rapid growth culminating in overweight/obesity by age 5 years.

## Methods

### Study design

The CHILD Cohort Study is a population-based prospective birth cohort study in Canada. It recruited 3621 pregnant mothers across four sites in Canada (Vancouver, Edmonton, Manitoba, and Toronto) between 2008 and 2012, from which 3455 delivered healthy, full-term infants, and were eligible to commence the study. The CHILD Cohort Study has been described in detail elsewhere [21]. This study was approved centrally by the Hamilton Integrated Research Ethics Board (HiREB #07-2929) and all local ethics boards, and informed consent was obtained from a parent or legal guardian.

### Anthropometric measurements

Birthweight of all children was collected from birth charts. All subsequent weight and height measurements were performed by trained research assistants using standardized procedures, at a home visit when children were 3 months of age, and at clinic visits when children were 1, 3 and 5 years old. Shoes and outerwear were removed for weight (Scaletronix scale) and height (standard stadiometer) measurements.

BMI at each time point was calculated and age and sex BMI *z*-scores (BMIz) were derived according to the World Health Organization (WHO) child growth standards for children younger than 5 years [22], and for 5–19 years for those children that were slightly older than 5 years old at the date of measurement [23].

### Growth trajectories

Growth trajectories were identified by group-based trajectory models (GBTM) and latent class mixed models (LCMM). Exact age at the time of measurement (and gestational age at birth) and BMIz at birth, 3 months, 1, 3 and 5 years were included in the analysis. Modeling was restricted to subjects with measurements available for at least three of the five time points, and was performed separately for boys and girls. Model performance was evaluated from two to eight trajectories. Optimal number of classes was chosen based on Bayes information criteria (BIC), Akaike information criteria (AIC), Log Bayes Factor (2log_e_(B_10_)), median posterior probability of assignment of at least 0.70, and on trajectories being distinct and interpretable. After comparison of trajectories between models, GBTM classifications were used in all subsequent analyses. Details on trajectory modeling can be found in the Supplementary Material.

### Stool sample collection and microbiota analysis

Fecal samples were collected at a home visit (3–4 months; mean [SD], 3.7 [1.1] months) and a clinic visit (12 months; mean [SD], 12.5 [1.7] months); DNA was extracted using the commercial kits (Qiagen Mo Bio PowerSoil) optimized for the Thermofisher KingFisher® robot.

Our approach to defining the gut microbiota of infants of the CHILD cohort has been previously described [24, 25]. Briefly, the V4 hypervariable region of the 16S rRNA gene of fecal DNA was amplified by PCR using universal bacterial primers (V4-515f: V4-806r). Paired-end sequences were pre-processed using Dada2 in Qiime2 v.2018.6 (www.qiime2.org). Taxonomic identity was assigned to the resulting Amplicon Sequence Variants by alignment to the Greengenes reference (v13.8) database at 99% sequence similarity. Sequences were further filtered to remove those that were present at less than 0.005% of the total sequences. Demultiplexed sequencing data used in this study are deposited into the Sequence Read Archive of NCBI and can be accessed via accession numbers PRJNA657821.

Samples were rarefied to 8000 sequencing reads per sample prior to computing all diversity metrics. Gut microbiota α-diversity was measured by Shannon index. Changes in microbiota diversity over time were calculated in paired samples as the difference between 3 months and 1 year of age. Analyses were completed in R software, version 4.0.2.

### Statistical analyses

Differences in continuous measures between weight trajectories are presented as median [Interquartile range] and evaluated by Wilcoxon rank test or Kruskal–Wallis test were appropriate. Differences in categorical variables are presented as frequencies (%) and evaluated by Pearson’s Chi-square test, post hoc tests for multiple comparisons of groups were performed by Dunn test. *p* values were adjusted for multiple comparisons using the Benjamini–Hochberg procedure.

After identification of BMIz trajectories, adjusted odds ratios (OR) and 95% confidence intervals (CI) from weighted multinomial regression were used to examine factors associated with each trajectory. Weights were based on individual posterior probabilities. All models were adjusted for prenatal smoke exposure, self-reported ethnicity (Caucasian vs. non-Caucasian), study site, and postpartum maternal BMI (measured when the child was 1 year of age). Next, the relationship between significant risk factors from adjusted models in relation to BMIz trajectories and the gut microbiota was analyzed by Kruskal–Wallis test for categorical factors and Spearman correlation for continuous factors.

To identify potential bacterial genera driving significant differences between trajectories, DESeq2 on raw count data with relative log expression normalization was applied, models were adjusted by self-reported ethnicity and study site. Genera that statistically discriminated between children with normative and high stable or rapid growth trajectories were selected. Ordinal logistic regression adjusted for ethnicity and study site was applied to analyze diversity trends of the selected genera across BMIz trajectories. Changes in genera with significant trends were further contrasted to the rapid growth trajectory.

## Results

### Growth trajectories

**Fig. 1:**
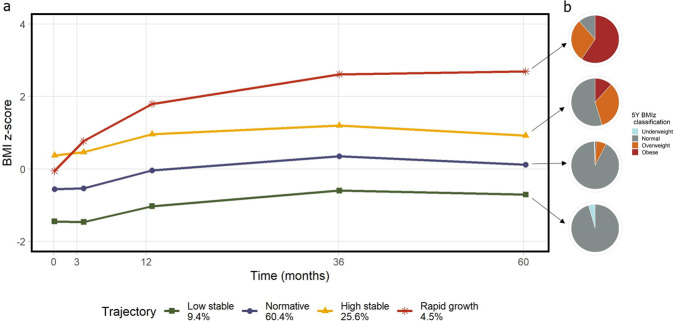
BMIz trajectories in the CHILD Cohort Study. **a** Predicted BMI *z*-score trajectories obtained from group-based trajectory modeling, and **b** proportion of overweight, obese children at 5 years per World Health Organization classification of BMI *z*-scores.

We modelled BMIz trajectories for 3059 participants within the CHILD Study (53.1% boys) who had at least three BMIz measurements available between birth and age 5 years. BIC and AIC statistics improved with increased number of classes in both LCMM and GBTM. The four-trajectory model from GBTM was favored based on approximation to the log of the Bayes factor, and median membership probability >0.70 for all classes in both boys and girls (Supplementary Table 1). Trajectories from both sexes had similar trends over time and were pooled together into one dataset for further analyses (Supplementary Fig. 1). The four trajectories were designated: low stable, normative, high stable and rapid growth (Fig. 1).

The normative trajectory contained 60% of participants (*n* = 1849), mean BMIz in this group started at −0.5 BMIz and trended toward zero by age 5 years. A total of 289 (9.4%) participants were placed in the low-stable weight trajectory, which followed a steady trend of −1 *z*-scores up to 5 years of age. The high stable trajectory had 782 (25.6%) participants and a mean BMIz of 0.5 up to 3 months, which increased to 1 BMIz at age 1 year. By age 3 years, 12% of subjects in this trajectory were classified as overweight, and 0.7% as obese. Finally, the rapid growth trajectory contained 139 (4.5%) participants and was characterized by a rapid increase in BMIz from birth, with a mean over 2.5 BMIz by age 3 years. Over 70% children in the rapid growth trajectory were overweight or obese by age 3 years, and 89% by age 5 years (Fig. 1).

**Fig. 2:**
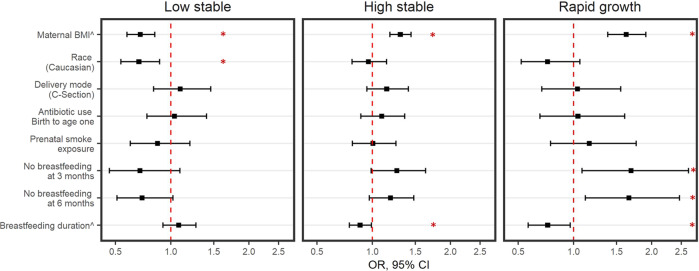
Adjusted odds ratio and 95% CI for the associated risk of individual factors on BMIz trajectories from weighted multinomial regression (normative trajectory used as reference group). All models adjusted for maternal BMI, prenatal smoke exposure, study site, and race. ^Odds ratio per interquartile increase of exposure variable (in maternal postpartum BMI and breastfeeding duration models).

We analyzed familial and environmental risk factors of early-life overweight and obesity to identify whether they were significantly associated to a certain BMIz trajectory. Adjusted models showed that children not breastfed at 3 or 6 months of age had 70% higher odds of belonging to the rapid growth trajectory, compared to the normative trajectory (3 months BF aOR 1.70, 95% 1.09, 2.64; 6 months BF aOR 1.67, 95% CI 1.13, 2.47) while increased duration of breastfeeding (in months) was associated with decreased risk of rapid growth (Fig. 2). We observed similar associations in the high stable trajectory, although only nominally significant for both 3 months (aOR 1.27, 95% CI 0.99, 1.64) and 6 months breastfeeding (aOR 1.20, 95%CI 0.97, 1.48). Increase in maternal BMI was significantly associated with 31% higher risk of belonging to the high stable trajectory (interquartile aOR 1.31, 95% CI 1.19, 1.45), and 63% for the rapid growth trajectory (aOR 1.63, 95%1.39, 1.91). Prenatal smoke exposure, delivery mode, reported antibiotic use in the first year of life, and race were not significantly associated to BMIz trajectories (Fig. 2 and Supplementary Table 2).

### Growth trajectories and gut microbiota diversity

In order to determine whether components of the infant microbiota differed among BMIz trajectories, we utilized results from 988 participants with stool collected at 3 months (*n* = 826) and/or 1 year (*n* = 842). A total of 680 participants contained paired data from both timepoints. The microbiota subgroup was similar to those in the overall cohort, including the proportion of subjects in each BMIz trajectory (Table 1 and Supplementary Table 3).

**Table 1:** Characteristics of all participants (*N* = 3059) in the CHILD Cohort Study and by BMIz trajectory groups.

	Overall Cohort (*N* = 3059)	Low-stable (*N* = 289, 9.4%)	Normative (*N* = 1849, 60.4%)	High stable (*N* = 782, 25.6%)	Rapid growth (*N* = 139, 4.5%)
Sex (Male)	1624 (53.1)	167 (57.8)	973 (52.6)	448 (57.3)	36 (25.9)
Race (Caucasian)	1957 (64.7)	158 (55.2)	1204 (65.8)	510 (65.9)	85 (63.0)
Delivery mode (Vaginal)	2253 (74.6)	224 (77.5)	1378 (76.0)	550 (70.3)	101 (72.7)
Gestational age (Months)	9.10 (0.31)	9.08 (0.32)	9.08 (0.32)	9.15 (0.28)	9.10 (0.30)
Prenatal smoke exposure (Yes)	547 (18.2)	41 (14.9)	322 (17.7)	151 (19.7)	33 (24.6)
Maternal BMI (kg/m^2^)	25.38 (5.86)	23.29 (4.60)	24.94 (5.48)	26.54 (6.33)	29.07 (7.53)
Antibiotic use—birth to age 1	573 (19.4)	51 (18.1)	338 (19.0)	154 (20.1)	30 (22.4)
Breastfeeding duration (Months)	10.70 (6.82)	12.19 (6.36)	11.04 (6.90)	9.71 (6.55)	8.73 (6.98)
Breastfeeding at 3 months					
Exclusive	1775 (60.2)	190 (67.6)	1113 (62.8)	410 (53.7)	62 (47.7)
Partial	768 (26.1)	70 (24.9)	437 (24.7)	224 (29.3)	37 (28.5)
None	404 (13.7)	21 (7.5)	222 (12.5)	130 (17.0)	31 (23.8)
Breastfeeding at 6 months					
Exclusive	530 (18.4)	56 (20.2)	351 (20.3)	110 (14.6)	13 (10.2)
Partial	1694 (58.7)	184 (66.4)	1009 (58.4)	437 (58.1)	64 (50.4)
None	661 (22.9)	37 (13.4)	369 (21.3)	205 (27.3)	50 (39.4)
Annual family income					
<$50K	358 (13.3)	29 (12.0)	213 (13.0)	91 (13.1)	25 (20.8)
$50K–<$100K	915 (34.0)	89 (36.9)	552 (33.7)	230 (33.2)	44 (36.7)
$100K–<$150K	756 (28.1)	59 (24.5)	460 (28.1)	207 (29.9)	30 (25.0)
≥$150K	661 (24.6)	64 (26.6)	411 (25.1)	165 (23.8)	21 (17.5)
BMI-z classification at 3 years					
Underweight	22 (0.8)	15 (5.6)	6 (0.4)	1 (0.1)	0 (0.0)
Normal range	2562 (92.1)	255 (94.4)	1651 (98.6)	621 (87.2)	35 (28.0)
Overweight	166 (6.0)	0 (0.0)	16 (1.0)	85 (11.9)	65 (52.0)
Obese	32 (1.2)	0 (0.0)	2 (0.1)	5 (0.7)	25 (20.0)
BMI-z classification at 5 years					
Underweight	24 (0.9)	12 (4.5)	12 (0.7)	0 (0.0)	0 (0.0)
Normal range	2159 (79.1)	253 (95.5)	1516 (91.7)	376 (54.7)	14 (11.6)
Overweight	379 (13.9)	0 (0.0)	112 (6.8)	232 (33.7)	35 (28.9)
Obese	166 (6.1)	0 (0.0)	14 (0.8)	80 (11.6)	72 (59.5)

**Fig. 3:**
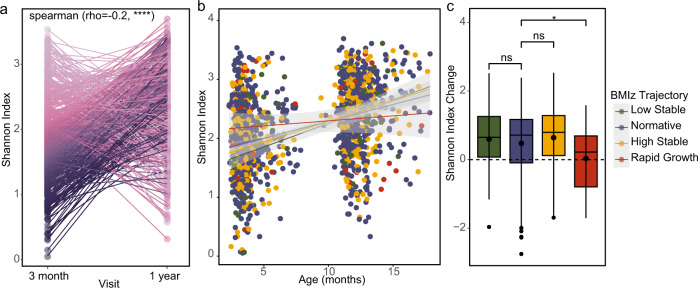
Alpha diversity of the gut microbiome over time in CHILD Cohort Study infants. **a** Paired Shannon index measures at 3 months and 1 year and corresponding Spearman correlation. Darker color indicates lower diversity at 3 months. **b** Shannon index over time by BMI-z trajectory groups. **c** Boxplot of changes in Shannon index between paired 3 months and 1 year samples. *p* values calculated from Wilcoxon rank-sum tests. ****p value <0.0001, *p value <0.05, ns = *p* value >0.05.

Previous studies have demonstrated the importance of bacterial diversity for maintaining healthy weight in both humans and mouse models, and much of this diversification of the microbiota occurs during the first year of life as infants are exposed to new colonizing microbes [7, 26, 27]. A published analysis from the CHILD study linked increased diversity at 3 months with greater risk of weight gain [28]. Supporting this, we found a significantly negative correlation between diversity at 3 months and diversity at 1 year (Spearman, *ρ* = −0.2, *p* < 0.0001) (Fig. 3a). Thus, early diversification may limit overall diversity at 1 year. Moreover, after quantifying changes in bacterial diversity over time, we observed that infants in the rapid growth trajectory gained less diversity (as measured by Shannon Index) between 3 months and 1 year of age compared to the normative BMIz trajectory (Fig. 3b, c). These differences in diversity were not significant at either 3 months (*p* = 0.10) or 1 year (*p* = 0.65) time points alone, but instead represent a cumulative change in diversification within the gut and emphasize the value of longitudinal sampling.

**Fig. 4:**
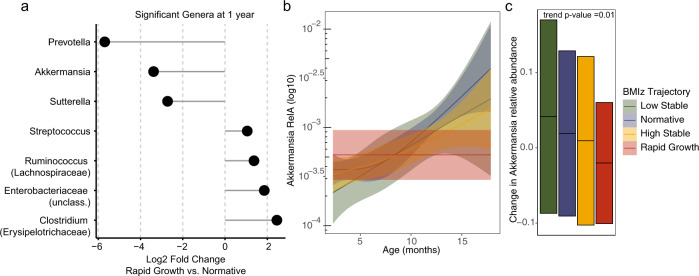
Differential abundance analysis on BMIz trajectories in the CHILD Cohort Study. **a** Log2 fold change of differential abundance at 1 year visit between normative and rapid growth trajectory. Only significant genera are shown. **b** Akkermansia relative abundance over time in each BMI-z trajectory. **c** Crossbar plots of mean and standard deviation reflecting change in relative abundance for Akkermansia between 3 months and 1 year in each BMI-z trajectory. *p* value corresponds to coefficient across BMIz trajectories from ordinal logistic regression adjusted for ethnicity and study site.

We performed a differential abundance analysis to understand whether the reduced diversification observed in the rapid growth trajectory coincided with changes in specific bacterial taxa. A number of genera were significantly altered at the 1 year visit, but none at the 3 months visit, including *Akkermansia*, unclassified *Enterobacteriaceae* members, *Clostridium*, *Sutterella*, and *Ruminococcus* (Fig. 4a).

To understand whether any of the observed taxa may be associated with the other weight trajectories in these children, we performed ordinal logistic regression, including all BMI-z trajectory groups as outcome and the change in abundance of each of the genera above. Only the relative abundance of *Akkermansia* had a significantly decreasing trend at 1 year (*p* = 0.01) and across time (*p* = 0.009) between the four BMIz trajectories (Fig. 4b, c). This was accompanied by a reduction in overall colonization accumulation between 3 months and 1 year in the rapid growth trajectory, suggesting that *Akkermansia* abundance may have a direct impact on overall BMIz gaining trajectories beginning in infancy.

### Gut microbiota diversity and risk factors for rapid growth

**Fig. 5:**
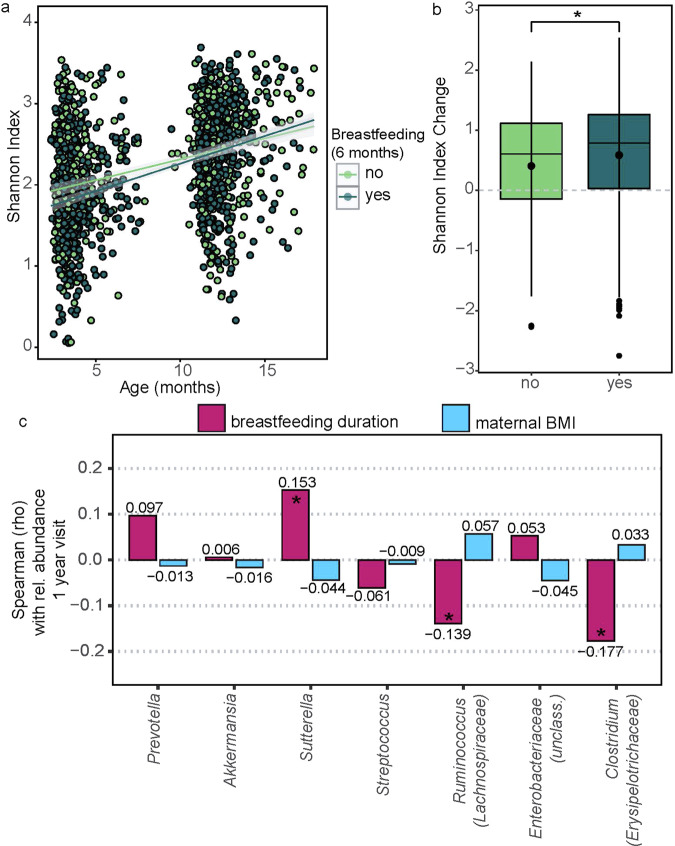
Gut microbiome diversity, composition and risk factors for rapid growth. **a** Shannon index over time by breastfeeding status at 6 months. **b** Boxplot of changes in Shannon index between paired 3 months and 1 year samples by breastfeeding status at 6 months. **c** Spearman-rank correlation of maternal BMI or breastfeeding duration and relative abundance of genera associated with rapid growth.

Maternal BMI and breastfeeding during early life were both associated with BMIz trajectories, and recent studies have implicated these in influencing the early-life microbiota composition as well [24, 29, 30]. We therefore investigated whether maternal BMI, breastfeeding duration and presence of breastfeeding were associated with microbiota diversification or changes in taxonomic relative abundance related to BMIz trajectories. Breastfeeding at 6 months of age was significantly associated with increased diversification of the infant gut over the first year of life (Fig. 5a, b). By comparison, postpartum maternal BMI was not associated with diversification (data not shown). Influences of breastfeeding were also seen on relative abundance of genera associated with rapid growth at 1 year of age (Fig. 5c). These include *Ruminococcus* and *Clostridium*, which were negatively correlated with breastfeeding duration, and *Sutterella*, which increased with breastfeeding duration. We did not identify any significant correlations with maternal BMI and any of the genera associated with rapid growth.

## Discussion

In this study, we harnessed the power of the longitudinal CHILD Cohort Study to provide new insights into the relationship between the gut microbiota composition in infancy and growth trajectories beginning at birth and up to 5 years of life. Using a cohort of full-term, healthy children, we identified four BMIz trajectories that coincided with different rates of growth based on BMI z-scores: low stable, normative growth, high stable, and rapid growth from birth. Importantly, while our observed trajectories were not fully in line with clinical BMIz outcomes at 3 and 5 years of age (per WHO standards), they were similar to those observed in other studies, such as a consortium study of eight European birth cohorts [31], as well as a study of 1957 children from the Quebec Longitudinal Study of Child Development [32]. Furthermore, these trajectories were associated with familial and environmental factors previously reported in the literature [33–35]. Thus, the utilization of these growth trajectories that exist prior to obvious differences in BMIz allow for early identification of at-risk children during infancy in order to address modifiable factors involved in development of childhood obesity.

The associations between obesity and rapid weight gain in infancy and its tracking to subsequent obesity in later life are well established [2, 36, 37], however studies aiming to generate mechanistic insights into childhood obesity have focused on snap shots of children who are already experiencing clinical signs of metabolic disorders. Here we coupled growth trajectories that exist between birth and 5 years with changes in microbiota composition between ages 3 months and 1 year, when the microbiota is undergoing its most dynamic changes in diversity and colonization in response to environmental exposures and dietary changes. We found that infants in the rapid growth trajectory experienced significantly less diversification of their gut microbiota during the first year of life and displayed an altered microbiota composition by 1 year when compared to normative infants. Many of these genera have been identified in other human and mouse studies of obesity, however, whether some of these are contributors or bystanders of weight gain has been difficult to parse out [38, 39]. We therefore investigated whether the colonization of any of these genera trended over all growth trajectories. In particular, the expansion of *Akkermansia*, the genus predominantly comprised of *Akkermansia muciniphila*, was blunted throughout the first year of life in infants experiencing rapid BMIz gain and displayed a significantly inverse trend in expansion with overall BMIz gain. Our findings that a significant overlap exists between *Akkermansia* abundance and patterns of growth supports a mechanistic link between this commensal and longitudinal weight gains that exist prior to obesity and may be sensitive to modification during infancy. Notably, *Akkermansia* is currently being investigated as a live biotherapeutic product, which may complement current family-based life style interventions that have shown only modest effect on weight loss [40].

In accordance to previous studies, we found that breastfeeding status and duration were significantly associated with different BMIz trajectories, particularly in the rapid growth trajectory [33]. Previous studies observed a dominant influence of breastfeeding on the infant microbiome when compared to maternal BMI, and our findings support this [29]. While we did not identify correlation between maternal BMI and the infant microbiota composition, we did observe significant associations between occurrence and duration of breastfeeding and both diversification and the relative abundance of genera that were increased within the rapid growth trajectory. Moreover, our finding that microbiota diversity at 3 months was higher in non-breastfed children aligns with that of a separate subset of 1087 children from the CHILD cohort published previously [28]. Similar to our findings, Forbes et al. additionally showed that infants who were overweight or at risk of being overweight at 1 year of age (as defined per WHO weight for length *z*-scores) had significantly higher richness of microbiota at 3 months of age [28], although the association was not significant in our analysis. While they postulated that increased energy extraction from a more diverse gut early in life increases the risk of being overweight at 1 year, their analysis did not include the longitudinal impact of early diversification on the microbiota. Indeed, our analyses indicate that early, high diversity may impede newly colonizing bacteria within the first year and limit total diversification. While we did not find direct associations between *Akkermansia* and breastfeeding duration, our analyses did not incorporate the heterogeneity of breastmilk metabolites, which have been demonstrated to influence *Akkermansia* in animal models [14]. Furthermore, *Akkermansia* expansion in our study was weakly correlated with diversification (spearman: *ρ* = 0.27, *p* = 4.6e−11), suggesting that they are intertwined.

Following the definitions used in Forbes et al. in our study sample, 62% (*n* = 412) and 22% (*n* = 147) of children classified as overweight or at risk of overweight at 1 year were placed into the high stable and normative trajectories, respectively. The remaining 16% (*n* = 106) of children were classified in the rapid growth trajectory in which we observed most relevant changes in microbiota. The fundamental differences in our findings may suggest that although overweight measures restricted to the infancy period may point to specific perturbations of the microbiota in early life, the identification of longitudinal phenotypes of obesity described in this study provide additional information about the role of the infant microbiota in preschool obesity that could otherwise be missed.

The CHILD cohort is unique in that it comprises a general population of multi-ethnic children, but considering it is an observational study, it is important to address the limitations of our study. Despite the clear associations between early-life BMI and chronic co-morbidities presented in other studies [5], there is ongoing debate as to the utility of BMI as an indicator of adiposity in infancy. Future studies should analyze gut microbiota regulation in early life with respect to body composition measures. Secondly, to minimize the inherent limitations of mixture modeling for identifying trajectories of growth, we relied on statistical criteria for class enumeration, performed stratified modeling by sex, and incorporated posterior probabilities into regression modeling [41]. Nevertheless, the associations reported here need to be validated by experimental models and replicated in independent human populations.

The burden in quality of life, mental and physical health as early as preschool age due to childhood obesity co-morbidities are well documented, but the underlying mechanisms and the role of the microbiota maturation in childhood obesity regulation remain unclear. This study provides evidence of the underlying biological alterations in children with different BMIz trajectories over time. The most striking associations were found in those children with a steep increase in BMI during the first year of life that continues up to 5 years of age. Our findings support a novel avenue of research on the human microbiota, targeted on tangible interventions to reduce childhood overweight and obesity.

## Supplementary information

Supplemental material

## Data Availability

A list of variables available in the CHILD Cohort Study is available at https://childstudy.ca/for-researchers/study-data/. Researchers interested in collaborating on a project and accessing CHILD Cohort Study data should contact the Study’s National Coordinating Centre (NCC) to discuss their needs before initiating a formal request. To contact the NCC, please email child@mcmaster.ca. More information about data access for the CHILD Cohort Study can be found at https://childstudy.ca/for-researchers/data-access/.

## References

[1] Tackling obesity in Canada: Childhood obesity and excess weight rates in Canada—Canada.ca [Internet]. 2021. https://www.canada.ca/en/public-health/services/publications/healthy-living/obesity-excess-weight-rates-canadian-children.html#shr-pg0.

[2] Smego A, Woo JG, Klein J, Suh C, Bansal D, Bliss S (2017). High body mass index in infancy may predict severe obesity in early childhood. J Pediatr.

[3] Garden FL, Marks GB, Simpson JM, Webb KL (2012). Body mass index (BMI) trajectories from birth to 11.5 years: relation to early life food intake. Nutrients..

[4] van Rossem L, Wijga AH, Brunekreef B, de Jongste JC, Kerkhof M, Postma DS (2014). Overweight in Infancy: Which Pre- and Perinatal Factors Determine Overweight Persistence or Reduction A Birth Cohort Followed for 11 Years. Ann Nutr Metab.

[5] Sharma V, Coleman S, Nixon J, Sharples L, Hamilton‐Shield J, Rutter H (2019). A systematic review and meta-analysis estimating the population prevalence of comorbidities in children and adolescents aged 5 to 18 years. Obes Rev.

[6] Ziyab AH, Karmaus W, Kurukulaaratchy RJ, Zhang H, Arshad SH (2014). Developmental trajectories of Body Mass Index from infancy to 18 years of age: prenatal determinants and health consequences. J Epidemiol Community Health.

[7] Petersen C, Bell R, Klag KA, Lee S-H, Soto R, Ghazaryan A (2019). T cell-mediated regulation of the microbiota protects against obesity. Science.

[8] Bäckhed F, Ding H, Wang T, Hooper LV, Koh GY, Nagy A (2004). The gut microbiota as an environmental factor that regulates fat storage. Proc Natl Acad Sci USA.

[9] Kim KN, Yao Y, Ju SY (2019). Short chain fatty acids and fecal microbiota abundance in humans with obesity: a systematic review and meta-analysis. Nutrients.

[10] Sender R, Fuchs S, Milo R (2016). Revised estimates for the number of human and bacteria cells in the body. PLoS Biol.

[11] Macchione IG, Lopetuso LR, Ianiro G, Napoli M, Gibiino G, Rizzatti G (2019). Akkermansia muciniphila: key player in metabolic and gastrointestinal disorders. Eur Rev Med Pharmacol Sci.

[12] Schneeberger M, Everard A, Gómez-Valadés AG, Matamoros S, Ramírez S, Delzenne NM (2015). Akkermansia muciniphila inversely correlates with the onset of inflammation, altered adipose tissue metabolism and metabolic disorders during obesity in mice. Sci Rep.

[13] Depommier C, Everard A, Druart C, Plovier H, Van Hul M, Vieira-Silva S (2019). Supplementation with Akkermansia muciniphila in overweight and obese human volunteers: a proof-of-concept exploratory study. Nat Med.

[14] Ribo S, Sánchez-Infantes D, Martinez-Guino L, García-Mantrana I, Ramon-Krauel M, Tondo M (2021). Increasing breast milk betaine modulates *Akkermansia* abundance in mammalian neonates and improves long-term metabolic health. Sci Transl Med.

[15] White RA, Bjørnholt JV, Baird DD, Midtvedt T, Harris JR, Pagano M (2013). Novel developmental analyses identify longitudinal patterns of early gut microbiota that affect infant growth. PLoS Comput Biol.

[16] Aris IM, Chen L-W, Tint MT, Pang WW, Soh SE, Saw S-M (2017). Body mass index trajectories in the first two years and subsequent childhood cardio-metabolic outcomes: a prospective multi-ethnic Asian cohort study. Sci Rep.

[17] Hemmingsson E (2018). Early childhood obesity risk factors: socioeconomic adversity, family dysfunction, offspring distress, and junk food self-medication. Curr Obes Rep.

[18] Mameli C, Mazzantini S, Zuccotti GV (2016). Nutrition in the first 1000 days: the origin of childhood obesity. Int J Environ Res Public Health.

[19] Tamburini S, Shen N, Wu HC, Clemente JC (2016). The microbiome in early life: implications for health outcomes. Nat Med.

[20] Petersen C, Turvey SE (2020). Can we prevent allergic disease? Understanding the links between the early life microbiome and allergic diseases of childhood. Curr Opin Pediatr.

[21] Subbarao P, Anand SS, Becker AB, Befus AD, Brauer M, Brook JR (2015). The Canadian Healthy Infant Longitudinal Development (CHILD) study: examining developmental origins of allergy and asthma. Thorax..

[22] De Onis M, editor. WHO child growth standards: length/height-for-age, weight-for-age, weight-for-length, weight-for-height and body mass index-for-age; methods and development. Geneva: WHO Press; 2006. p. 312.

[23] de Onis M (2007). Development of a WHO growth reference for school-aged children and adolescents. Bull World Health Organ.

[24] Fehr K, Moossavi S, Sbihi H, Boutin RCT, Bode L, Robertson B (2020). Breastmilk feeding practices are associated with the co-occurrence of bacteria in mothers’ milk and the infant gut: the CHILD cohort study. Cell Host Microbe.

[25] Patrick DM, Sbihi H, Dai DLY, Al Mamun A, Rasali D, Rose C (2020). Decreasing antibiotic use, the gut microbiota, and asthma incidence in children: evidence from population-based and prospective cohort studies. Lancet Respir Med.

[26] Le Chatelier E, Nielsen T, Qin J, Prifti E, Hildebrand F, Falony G (2013). Richness of human gut microbiome correlates with metabolic markers. Nature..

[27] Lim ES, Zhou Y, Zhao G, Bauer IK, Droit L, Ndao IM (2015). Early life dynamics of the human gut virome and bacterial microbiome in infants. Nat Med.

[28] Forbes JD, Azad MB, Vehling L, Tun HM, Konya TB, Guttman DS (2018). Association of exposure to formula in the hospital and subsequent infant feeding practices with gut microbiota and risk of overweight in the first year of life. JAMA Pediatr.

[29] Stewart CJ, Ajami NJ, O’Brien JL, Hutchinson DS, Smith DP, Wong MC (2018). Temporal development of the gut microbiome in early childhood from the TEDDY study. Nature..

[30] Gohir W, Ratcliffe EM, Sloboda DM (2015). Of the bugs that shape us: maternal obesity, the gut microbiome, and long-term disease risk. Pediatr Res.

[31] Rzehak P, Wijga AH, Keil T, Eller E, Bindslev-Jensen C, Smit HA (2013). Body mass index trajectory classes and incident asthma in childhood: results from 8 European Birth Cohorts—a Global Allergy and Asthma European Network initiative. J Allergy Clin Immunol.

[32] Pryor LE (2011). Developmental trajectories of body mass index in early childhood and their risk factors: an 8-year longitudinal study. Arch Pediatr Adolesc Med.

[33] Azad MB, Vehling L, Chan D, Klopp A, Nickel NC, McGavock JM (2018). Infant feeding and weight gain: separating breast milk from breastfeeding and formula from food. Pediatrics..

[34] Stuart B, Panico L (2016). Early-childhood BMI trajectories: evidence from a prospective, nationally representative British cohort study. Nutr Diabetes.

[35] Mattsson M, Maher GM, Boland F, Fitzgerald AP, Murray DM, Biesma R (2019). Group‐based trajectory modelling for BMI trajectories in childhood: a systematic review. Obes Rev.

[36] Butler ÉM, Pillai A, Morton SMB, Seers BM, Walker CG, Ly K (2021). A prediction model for childhood obesity in New Zealand. Sci Rep.

[37] Das T, Ostbye T, Chan YH, Tan NC, Chew E (2021). Maternal and infant predictors of growth trajectories in singapore children in the first 18 months of life. BMJ Pediatrics Open..

[38] Mbakwa CA, Hermes GDA, Penders J, Savelkoul PHM, Thijs C, Dagnelie PC (2018). Gut microbiota and body weight in school-aged children: the KOALA Birth Cohort Study. Obesity.

[39] Tun MH, Tun HM, Mahoney JJ, Konya TB, Guttam DS, Becker AB (2018). Postnatal exposure to household disinfectants, infant gut microbiota and subsequent risk of overweight in children. CMAJ..

[40] Kumar S, Kelly AS (2017). Review of childhood obesity: from epidemiology, etiology, and comorbidities to clinical assessment and treatment. Mayo Clin Proc.

[41] van der Nest G, Lima Passos V, Candel MJJM, van Breukelen GJP (2020). An overview of mixture modelling for latent evolutions in longitudinal data: modelling approaches, fit statistics and software. Adv Life Course Res.

