# Supplementary material for: Longitudinal body mass index trajectories at preschool age: children with rapid growth have differential composition of the gut microbiota in the first year of life
Source: Int J Obes (Lond). 2022 Apr 15;46(7):1351–8. doi: 10.1038/s41366-022-01117-z (PMC9239911; doi:10.1038/s41366-022-01117-z)
Supplement: Supplementary file 1 — Supplemental material [file 41366_2022_1117_MOESM1_ESM.docx]

**Supplemental Material**

**Longitudinal Body Mass Index Trajectories at Preschool Age: Children with Rapid Growth have Differential Composition of the Gut Microbiota in the First Year of Life**

Myrtha E. Reyna*, MSc^a^, Charisse Petersen*, PhD^b^, Darlene L.Y. Dai^b^, Ruixue Dai, MSc^a^, Kozeta Miliku, MD PhD^f^, MSc^a^, Diana L. Lefebvre PhD^f^, Allan B. Becker, MD^c,d^, Meghan B. Azad, PhD^c,d,e^, Theo J. Moraes, MD, PhD^a^, Piushkumar J. Mandhane, MD, PhD^g^, Malcolm R. Sears, MB, ChB^f^, Brett B Finlay, PhD^i,j^, Elinor Simons, MD, PhD^c^, Anita L. Kozyrskyj, PhD^g^, Wendy Lou, PhD^h^, Stuart E. Turvey**, MBBS, DPhil^b^, Padmaja Subbarao** MD, MSc^a,f,h^.

**Corresponding author**

Dr. Padmaja Subbarao, MD MSc

The Hospital for Sick Children

555 University Avenue, Toronto, Canada M5G1X8

Email: padmaja.subbarao@sickkids.ca

Tel: (416) 813-2196

Fax: (416) 813-6246

**Methods**

*Growth trajectories*

Growth trajectories were identified by latent class growth analysis using group based trajectory modeling (GBTM), and by growth mixture models using latent class mixture models (LCMM). Modelling was restricted to subjects with measurements available for at least three of the five time points (N=3059), and was performed separately for boys and girls. Model performance was evaluated from two to eight trajectories.

BMI at each time point was adjusted for age and sex by calculating BMI z-scores (BMIz) according to the World Health Organization (WHO) child growth standards for children younger than 5 years, and for 5-19 years for those children that were slightly older than 5 years old at the date of measurement. Since WHO equations do not account for gestational age, the age at time of measurement included in trajectory models was calculated as the exact age in which BMI measurement plus the participants’ gestational age.

Optimal number of classes was chosen based on Bayes Information Criteria (BIC), Akaike Information Criteria (AIC), Bayes Factor, median posterior probability of assignment of at least 0.70 (i.e. the probability of each individual belonging to each trajectory group), and on trajectories being distinct and interpretable. In GBTM analysis, all models were tested with combinations of linear, quadratic and cubic time effects. In LCMM analysis models were tested using four equidistant splines. AIC and BIC improved with increased number of classes in both GBTM and LCMM, in both cases, the four trajectory models were favoured based on approximation to the log of the Bayes factor (2log_e_(B_10_)) and median membership probability >0.70 for all groups (Supplemental Table 1).

Participant placement in each trajectory was similar between GBTM and LCMM, with over 90% of participants placed in the same trajectory by both models, with the exception of the rapid growth group, where 56.1% of participants were classified differently. We observed that GBTM was more sensitive to identification of participants with rapid growth in the first year of life without compromising posterior probability, thus trajectories derived from GBTM were used in all subsequent analyses. We observed fourteen individuals (11.6%) in the rapid growth trajectory with normal BMIz (per WHO standards) at 5 years. All of these fourteen children shared a steep increase in BMIz after birth that only started decreasing after 1 or 3 years of age.

GBTM analyses were performed in SAS version 9.4 using the PROC TRAJ procedure, and LCMM analyses were performed in R version 4.0.2 and the LCMM package. All codes used in trajectory analysis are available upon request to the authors (Myrtha.reynavargas@sickkids.ca).

**Supplemental tables and figures**

Supplemental Table 1. Model diagnostics from group based trajectory models with 2 to 6 classes in the CHILD Cohort Study.

|  | **Boys (N=1624)** | | | | **Girls** | | | |
| --- | --- | --- | --- | --- | --- | --- | --- | --- |
| **Model** | **AIC BIC** | **BF_10_** | **N (%)** | **Median posterior probability** | **AIC BIC** | **BF_10_** | **N** | **Median posterior probability** |
| Two  classes | -10358.7  -10379.7 | - | 1071 | 0.86 | -8847.4 -8874.4 | - | 805 | 0.96 |
|  |  |  | 553 | 0.79 |  |  | 630 | 0.95 |
| Three  classes | -10231.6 -10279.7 | 212 | 774 | 0.93 | -8747.16 -8791.05 | 166.8 | 263 | 0.84 |
|  |  |  | 803 | 0.91 |  |  | 909 | 0.87 |
|  |  |  | 47 | 0.97 |  |  | 263 | 0.9 |
| **Four**  **classes** | **-10176.4 -10238.3** | **82.9** | **167** | **0.79** | **-8702.98 -8763.75** | **54.6** | **122** | **0.85** |
|  |  |  | **973** | **0.86** |  |  | **876** | **0.89** |
|  |  |  | **448** | **0.86** |  |  | **334** | **0.77** |
|  |  |  | **36** | **0.97** |  |  | **103** | **0.75** |
| Five  classes | -10144.8 -10227.3 | 22 | 200 | 0.79 | -8645.81 -8726.83 | 73.8 | 82 | 0.86 |
|  |  |  | 172 | 0.60 |  |  | 163 | 0.66 |
|  |  |  | 453 | 0.85 |  |  | 434 | 0.79 |
|  |  |  | 762 | 0.74 |  |  | 666 | 0.83 |
|  |  |  | 37 | 0.98 |  |  | 90 | 0.84 |
| Six  classes | -10124.6 -10224.2 | 6.2 | 196 | 0.80 | -8618.23 -8716.13 | 21.4 | 74 | 0.89 |
|  |  |  | 185 | 0.59 |  |  | 168 | 0.66 |
|  |  |  | 726 | 0.73 |  |  | 606 | 0.82 |
|  |  |  | 473 | 0.85 |  |  | 463 | 0.76 |
|  |  |  | 18 | 0.83 |  |  | 103 | 0.75 |
|  |  |  | 26 | 0.99 |  |  | 21 | 0.96 |

Model diagnostics for seven and eight trajectory models not shown.

Supplemental Table 2. Adjusted odds ratio and 95% CI for the associated risk of individual factors on BMIz trajectories (Normative trajectory used as reference group). All models adjusted for maternal BMI, prenatal smoke exposure, race and study site and are weighted by individual posterior probability of placement in each trajectory.

|  | **Low stable growth** | **High stable** | **Rapid growth** |
| --- | --- | --- | --- |
| **Maternal BMI^**  (kg/m^2^) | 0.71 (0.59, 0.84)* | 1.31 (1.19, 1.45)* | 1.63 (1.39, 1.91)* |
| **Race**  (Caucasian vs Other) | 0.69 (0.54, 0.89)* | 0.96 (0.80, 1.15) | 0.75 (0.52, 1.07) |
| **Mode of delivery**  (Vaginal vs C-Section) | 1.10 (0.83, 1.46) | 1.16 (0.95, 1.41) | 1.04 (0.70, 1.56) |
| **Prenatal smoke exposure**  (Yes vs No) | 0.87 (0.62, 1.21) | 1.01 (0.81, 1.26) | 1.17 (0.77, 1.77) |
| **No breastfeeding (vs any) at 3 months** | 0.70 (0.45, 1.09) | 1.27 (0.99, 1.64) | 1.70 (1.09, 2.64)* |
| **No breastfeeding (vs any) at 6 months** | 0.72 (0.51, 1.02) | 1.20 (0.97, 1.48) | 1.67 (1.13, 2.47)* |
| **Breastfeeding duration^**  (months) | 1.08 (0.92, 1.28) | 0.88 (0.78, 0.99)* | 0.75 (0.58, 0.97)* |

^Odds Ratio per interquartile increase of exposure variable.

Supplemental Table 3. Characteristics of CHILD study subset with gut microbiota data (N=988) and by BMIz trajectory.

|  | | **Subset with microbiome data**  (N=988) | | **Low stable growth**  (N=83, 8.4%) | | | **Normative**  (N=592, 60.0%) | | | **High stable**  (N=270, 27.3%) | | | **Rapid**  **growth**  (N=43, 4.2%) |
| --- | --- | --- | --- | --- | --- | --- | --- | --- | --- | --- | --- | --- | --- |
| **Participants with gut microbiota data** | | | | |  | | |  | | | |  | |
| At 3 months of age | | 842 (50.5) | | 75 (52.4) | | | 498 (50.2) | | | 233 (50.4) | | | 36 (50.7) |
| At 1 year of age | | 826 (49.5) | | 68 (47.6) | | | 494 (49.8) | | | 229 (49.6) | | | 35 (49.3) |
| **Sex (Male)** | | 568 (57.5) | | 53 (63.9) | | | 330 (55.7) | | | 169 (62.6) | | | 16 (37.2) |
| **Race (Caucasian)** | | 634 (64.5) | | 45 (54.2) | | | 377 (64.0) | | | 186 (69.4) | | | 26 (60.5) |
| **Delivery mode (Vaginal)** | | 735 (75.2) | | 61 (73.5) | | | 452 (77.7) | | | 189 (70.0) | | | 33 (76.7) |
| **Prenatal smoke exposure (Yes)** | | 162 (16.6) | | 7 (8.6) | | | 96 (16.5) | | | 48 (17.9) | | | 11 (25.6) |
| **Maternal BMI (kg/m^2^)** | | 25.19 (5.60) | | 23.04 (3.95) | | | 24.81 (5.48) | | | 26.15 (5.89) | | | 28.74 (5.70) |
| **Antibiotic use - birth to age 1** | | 231 (23.4) | | 24 (28.9) | | | 132 (22.4) | | | 65 (24.1) | | | 10 (23.3) |
| **Months of breastfeeding** | | 10.79 (6.74) | | 11.41 (6.30) | | | 11.09 (6.83) | | | 10.22 (6.68) | | | 9.09 (6.38) |
| **Breastfeeding at 3 months** | |  | | |  | | |  | | | |  | |
| Exclusive | | 570 (57.8) | | 49 (59.0) | | | 362 (61.3) | | | 141 (52.2) | | | 18 (42.9) |
| Partial | | 281 (28.5) | | 26 (31.3) | | | 151 (25.5) | | | 87 (32.2) | | | 17 (40.5) |
| None | | 135 (13.7) | | 8 ( 9.6) | | | 78 (13.2) | | | 42 (15.6) | | | 7 (16.7) |
| **Breastfeeding at 6 months** | |  | | |  | | |  | | | |  | |
| Exclusive | | 167 (17.1) | | 12 (14.5) | | | 116 (19.8) | | | 34 (12.7) | | | 5 (12.2) |
| Partial | | 593 (60.6) | | 59 (71.1) | | | 344 (58.7) | | | 167 (62.3) | | | 23 (56.1) |
| None | | 218 (22.3) | | 12 (14.5) | | | 126 (21.5) | | | 67 (25.0) | | | 13 (31.7) |
| **Annual Family Income** |  | | | | |  | | | | |  | | |
| < $50K | | 116 (13.1) | | 11 (15.3) | | | 71 (13.5) | | | 26 (10.6) | | | 8 (19.5) |
| $50K - < $100K | | 302 (34.2) | | 30 (41.7) | | | 168 (32.1) | | | 87 (35.4) | | | 17 (41.5) |
| $100K - < $150K | | 271 (30.7) | | 15 (20.8) | | | 166 (31.7) | | | 78 (31.7) | | | 12 (29.3) |
| ≥ $150K | | 194 (22.0) | | 16 (22.2) | | | 119 (22.7) | | | 55 (22.4) | | | 4 (9.8) |
| **BMI-z classification at 3 years** | | |  | | | | | |  | | | | |
| Underweight | | 4 (0.4) | | 3 (3.7) | | | 1 (0.2) | | | 0 (0.0) | | | 0 (0.0) |
| Normal range | | 852 (91.5) | | 79 (96.3) | | | 549 (98.7) | | | 216 (85.4) | | | 8 (20.0) |
| Overweight | | 14 (1.5) | | 0 ( 0.0) | | | 1 (0.2) | | | 3 (1.2) | | | 10 (25.0) |
| Obese | | 61 (6.6) | | 0 ( 0.0) | | | 5 (0.9) | | | 34 (13.4) | | | 22 (55.0) |
| **BMI-z classification at 5 years** | | |  | | | | | |  | | | | |
| Underweight | | 8 (0.9) | | 6 (7.7) | | | 2 (0.4) | | | 0 (0.0) | | | 0 (0.0) |
| Normal range | | 704 (77.3) | | 72 (92.3) | | | 495 (91.3) | | | 134 (53.2) | | | 3 (7.7) |
| Overweight | | 62 (6.8) | | 0 ( 0.0) | | | 3 (0.6) | | | 34 (13.5) | | | 25 (64.1) |
| Obese | | 137 (15.0) | | 0 ( 0.0) | | | 42 (7.7) | | | 84 (33.3) | | | 11 (28.2) |


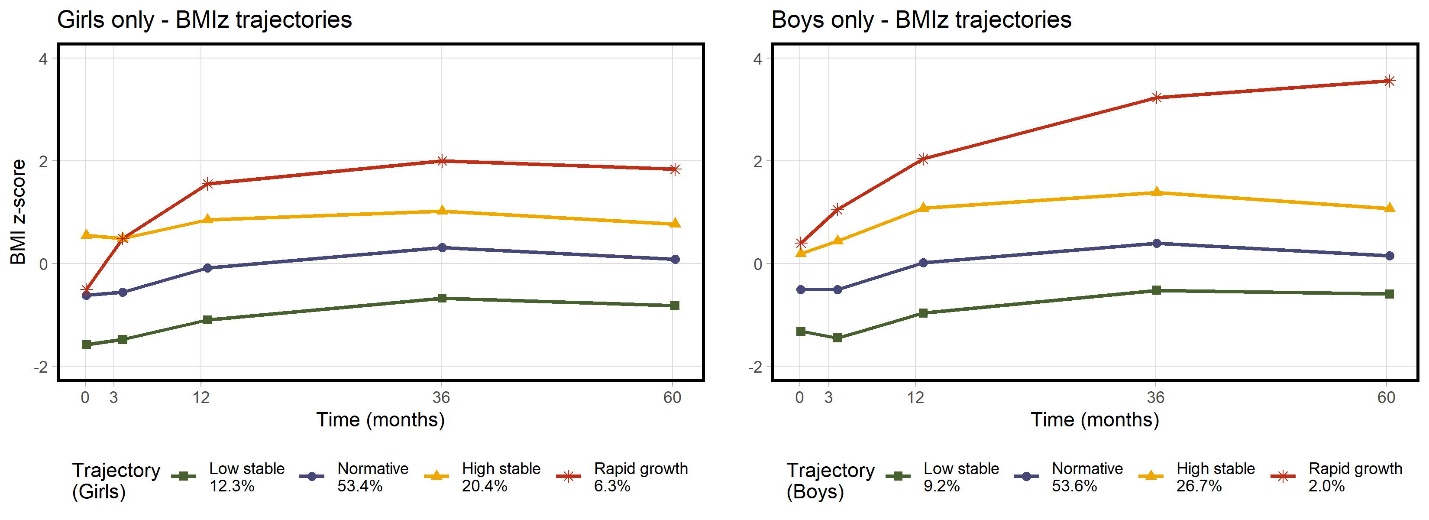


Supplemental Figure 1. Predicted BMI z-score trajectories obtained from stratified group based trajectory modelling by sex. Resulting trajectories had similar trends over time and were pooled together into one dataset as shown in Figure 1.
